# The Impact of Black Walnut (*Juglans nigra*) Oil on Male Wistar Rats Suffering from Methimazole-Induced Hypothyroidism

**DOI:** 10.5812/ijpr-161449

**Published:** 2025-08-19

**Authors:** Sedighe Morakabati, Alireza Motavalizadehkakhky, Amirhossein Esmaeili, Jamshid Mehrzad, Rahele Zhiani

**Affiliations:** 1 Department of Biochemistry, Ne.C., Islamic Azad University, Neyshabur, Iran; 2 Department of Chemistry, Ne.C., Islamic Azad University, Neyshabur, Iran; 3 Department of Laboratory Science, Bab.C., Islamic Azad University, Babol, Iran

**Keywords:** Hypothyroidism, Black Walnut, Malondialdehyde, Nitric oxide, Catalase, Superoxide Dismutase.

## Abstract

**Background:**

Hypothyroidism is a major endocrine disorder characterized by the dysfunction of thyroid hormones.

**Objectives:**

This study aimed to investigate the potential therapeutic effects of an oily extract derived from black walnut (*Juglans nigra*) kernels on methimazole-induced hypothyroidism in adult Wistar rats.

**Methods:**

In this experimental study, hypothyroidism was induced by the oral administration of methimazole at a single dose of 25 mg/kg body weight. In this research, 40 adult male rats were used in five groups of eight, all of which were administered orally. At the end of the experimental period, blood samples were collected from the hearts of animals for the evaluation of oxidative stress and thyroid function parameters. Serum malondialdehyde (MDA) and nitric oxide (NO) levels were assessed as markers of oxidative stress, while catalase (CAT) and superoxide dismutase (SOD) enzyme activities were measured as indicators of antioxidant defense. Thyroid hormone levels [triiodothyronine (T3), thyroxine (T4), and thyroid-stimulating hormone (TSH)] were determined using ELISA-based assay kits. Statistical analysis was performed using one-way ANOVA followed by Tukey’s post-hoc test.

**Results:**

Black walnut kernel oil was found to contain essential fatty acids, various hydrocarbons, and phenolic compounds. Consequently, using treatment with black walnut kernel extract in hypothyroidism rats, the SOD and T4 levels were decreased in the methimazole-treated stress group in comparison with the control group (P > 0.05), while T3 and CAT levels were significantly decreased in the methimazole-treated stress group in comparison with the control group (P < 0.05). Furthermore, the TSH, Anti thyroid peroxidase (TPO), NO, and MDA levels were increased in the methimazole-treated stress group in comparison with the control group (P > 0.05).

**Conclusions:**

Although the ethanolic extract of black walnut (*J. nigra*) led to an increase in T4 levels in hypothyroid rats, the change was not statistically significant, and T3 levels were significantly decreased. Therefore, the extract did not demonstrate a clear improvement in thyroid function.

## 1. Background

Hypothyroidism is a clinical syndrome caused by a deficiency of thyroid hormones. Clinically, hypothyroidism manifests as an increase in serum concentrations of thyroid-stimulating hormone (TSH) accompanied by decreased levels of free triiodothyronine (FT4) and free thyroxine (FT3) (1). Hypothyroidism is usually associated with symptoms such as slow brain activity, fatigue, muscle weakness, drowsiness, slow heart rate, reduced cardiac output, and decreased blood volume (2). It also causes disorders and clinical symptoms in all body systems, including the respiratory system, which is accompanied by symptoms of alveolar hypoventilation, hypercapnia, skeletal muscle myopathy, decreased pulmonary carbon monoxide diffusion, respiratory failure, and obstructive sleep apnea (3). The thyroid gland is one of the largest endocrine glands, and its hormones, thyroxine (T4) and triiodothyronine (T3), play an important role in regulating metabolism, growth, development, maturation, and reproduction. Thyroid hormones are essential for differentiation, growth, metabolism, and physiological functions across all body tissues (4, 5). Treatment with methimazole is a suitable method for inducing hypothyroidism. This substance is classified as a thionamide antithyroid medicine, which can control and treat hyperthyroidism. Methimazole inhibits the synthesis of thyroid hormones by blocking thyroid peroxidase (TPO) activity within the gland (6).

Free radicals are involved in the development and pathogenesis of some diseases. Antioxidants can prevent the formation of free radicals, scavenge them, or accelerate their destruction. Antioxidants are the first line of cellular defense against free radical damage and are crucial for maintaining good health and well-being (7). A common complication of hypothyroidism is an imbalance in the antioxidant system and increased oxidative stress (8). When the production of free radicals exceeds the capacity of the antioxidant defense system, it disrupts physiological homeostasis and contributes to cellular dysfunction (9). Oxidative stress is a pathological process that occurs because of an imbalance in the antioxidant defense systems. Furthermore, the antioxidant enzymes, including catalase (CAT) and superoxide dismutase (SOD), can protect the tissues from free radical damage by neutralizing them. The SOD converts superoxide free radicals into oxygen, deactivating their function. Peroxidation of unsaturated fatty acids is one of the most important pathological effects of free radicals. For instance, malondialdehyde (MDA) is a highly toxic biocompound, forming from oxidative damage to cell membrane lipids (10, 11). Black walnut, *Juglans nigra* L., belongs to the Juglandaceae family. Traditionally, black walnut has been used in herbal medicine for the treatment of various ailments, including stomachache, asthma, eczema, skin disorders, sinusitis, endocrine disorders, as well as infectious and parasitic diseases. It also exhibits notable antimicrobial and antioxidant properties (12). Different parts of the walnut, including the green skin on the walnut fruit, the bark, the kernel, and the walnut leaves, are used in traditional medicine and pharmacognosy (13, 14). The higher antioxidant phenolic contents and monounsaturated fatty acids of black walnut make it more valuable in terms of nutritional and medical aspects compared to Persian walnut (15), which is famous for its antioxidant activity resulting from its fatty acids, phenolic content, tannins, tocopherols, and indoleamine (16). Besides, tannin and the extract of black walnut kernel act as antibiotic agents, inhibiting the growth of *Staphylococcus aureus* (17, 18).

## 2. Objectives

Given these properties, we aimed to evaluate the therapeutic potential of the extract of black walnut (*J. nigra*) kernels in the treatment of methimazole-induced hypothyroidism in Wistar rats. Accordingly, we assessed the extract's effects on thyroid tissue and the oxidant-antioxidant factors in hypothyroid rats.

## 3. Methods

### 3.1. Ethical Statement and Animals

The study protocol followed the guidelines for the care and use of laboratory animals developed by the Ethics Committee at the Islamic Azad University, Neyshabur Branch, Iran. Forty adult male Wistar rats weighing 200 ± 20 g, purchased from the Pasteur Institute (Iran), were employed in this experiment. Female rats undergo hormonal fluctuations that can alter biochemical parameters and thyroid hormone levels. Therefore, in this study, male rats were used due to the absence of such hormonal changes. They were housed in transparent cages under standard lab conditions (temperature 23 ± 2°C, humidity 60 ± 4%, and a 12-hour cycle of light and darkness), with free access to standard rodent food and tap water ad libitum.

### 3.2. Extraction and Chemical Analysis

Fresh kernels of black walnut (100 g) were powdered, and the oil was extracted for 24 hours at room temperature with ethanol (250 mL, 96%) as the solvent. Using a Whatman^®^ microfilter (0.45 μm pore diameter), the resultant extract was filtered, and the ethanol was eliminated using a rotary evaporator (Buchi Rotavapor R-200) at 40°C for six hours. The chemical composition of the black walnut kernel oil was identified using gas chromatography-mass spectrometry (GC-MS) on an Agilent Technology 7890 Agus chromatograph MASS Spectrometer system (model 5975C, USA) outfitted with an HP-5MS column (30 m length, an internal diameter of 0.25 mm, and a 0.25 μm film thickness; Agilent Technologies, USA).

### 3.3. Total Phenolic Quantification

The total phenol concentration of the extract was determined using Folin-Ciocalteu's reagent and external calibration with gallic acid. In brief, 250 µL of filtered extract (10 mg/mL) was incubated at 37°C with 0.2 M Folin-Ciocalteu reagent (1.25 mL). After 5 minutes, 1 mL of Na_2_CO_3_ (10%) was incubated with the solution for two hours at room temperature. Using a UV-2100 spectrophotometer (UNICO America), the solution's optical density was observed at 760 nm compared to a blank. The total phenol concentration was reported as µg of gallic acid equivalents (GAE) per gram of dry extract (19). All assays were conducted in triplicate, and averages were reported.

### 3.4. Total Flavonoid Quantification

The total flavonoid concentration of the black walnut kernel extract was quantified with the aluminum chloride colorimetric assay (20). In brief, 250 µL of the walnut kernel extract (10 mg/mL) was incubated at 37°C in a mixture of methanol (750 µL), aluminum chloride 10% (50 µL), potassium acetate (1 M, 50 µL), and purified water (1.4 mL). After 30 minutes, the optical density compared to a blank was determined at 415 nm. The total flavonoid concentration was reported as µg of quercetin equivalents (QUE) per gram of dry extract. All assays were conducted in triplicate, and averages were reported.

### 3.5. Experimental Design

To induce hypothyroidism, methimazole was given to rats by gavage at a dose of 25 mg/kg body weight. The drug administration method was as follows: The walnut extract was prepared at a concentration of 1000 mg/kg body weight. It is necessary to pull the head skin back slightly to open the rat's mouth and leave the esophagus completely open. Then, the extract was inserted into the rat's mouth using a syringe equipped with a gavage needle. The needle should be easily inserted into the esophagus, and care should be taken to ensure that the extract is swallowed completely and does not come out of the mouth. The same method was also used to administer distilled water to the control group. In this study, the duration of feeding distilled water and walnut extract was considered to be 42 days, which was done daily between 8 - 9 am. The drug was administered orally at a fixed time of day. In animal grouping, the total number of rats used in this study was 40 (Table 1). Initially, these rats were weighed and placed into 5 groups of eight based on weight range.

**Table 1.** The Tested Groups in This Study

Groups	Purpose	Dosage (per kg)	Treatment	Description
**1**	Negative control	-	Water + Standard food	Healthy control
**2**	Positive disease model	25 mg/kg	Methimazole only	Hypothyroid (stress control)
**3**	Assess extract alone	1000 mg/kg	Walnut extract only	Walnut control
**4**	Assess therapeutic effect	25 mg/kg + 1000 mg/kg	Methimazole + Walnut	Treated with walnut after stress induction
**5**	Compare with standard therapy	25 mg/kg + 1.6 µg/kg	Methimazole + Levothyroxine	Standard drug treatment

Group 1or control group, which received only water and concentrated food during the study period. Group 2 or stress control group, which received about 25 mg of methimazole per kilogram of rat body weight dissolved in 1 mL of distilled water by oral gavage. Group 3 or control group receiving plant extract, which received only black walnut ethanolic extract at a rate of 1000 mg per kilogram of body weight daily and orally by gavage. Group 4 or stress group receiving plant extract, which received 25 mg per kilogram of body weight of methimazole, was initially stressed, then fed by walnut extract at a rate of 1000 mg per kilogram of body weight daily and orally by gavage. Group 5 or the stress group received levothyroxine. In this group, rats in the stress group were administered 0.1 mg levothyroxine tablets (manufactured by Iran Hormone Pharmaceutical Company) at a dosage of 1.6 micrograms per kilogram of body weight dissolved in 1 mL of water by daily gavage for 6 weeks. Twenty-four hours after the last administration, the animals were anesthetized, and blood was collected from their ventricles. Their serum was immediately separated and divided into separate vials, which were stored at -20°C until the time of measuring thyroid hormones and oxidant and antioxidant factors (21).

### 3.6. Serum Levels of Thyroid Hormones and Thyroid-Stimulating Hormone

To assess the levels of T4, T3, and TSH, an ELISA thyroid hormone assay kit manufactured by Pishgaman Sanjesh Isatis Company of Iran was used. In this ELISA test, a specific monoclonal antibody prepared against one of the antigenic indices of T4, T3, and TSH molecules was used (22). To assess the levels of thyroid peroxidase antibody (anti-TPO), an ELISA anti-TPO assay kit manufactured by Mybiosource Company of the United States was used. The optical density of serum specimens was observed at 450 nm by a microplate ELISA reader (Bio-Rad, USA).

### 3.7. Determination of Malondialdehyde Levels

The Wills method was used to evaluate the level of MDA. In this method, the amount of MDA in the serum sample was evaluated by the reaction of the sample with thiobarbituric acid (TBARS) using a spectrophotometer at a wavelength of 532 nm. Correspondingly, 2.5 mL of trichloroacetic acid solution was mixed with 0.5 mL of serum from the studied animal in a test tube, and the mixture was heated in a boiling bath for 15 minutes. Then, the tubes were cooled down to room temperature and centrifuged at 1000 g for 10 minutes at 4°C. Two mL of the supernatant obtained from the centrifugation was transferred into a new test tube, and 1 mL of TBARS acid solution was added to it. The tubes were again placed in the boiling bath for 15 minutes, and after cooling down to room temperature, their absorbance was evaluated against a blank at a wavelength of 523 nm (23).

### 3.8. Assessment of Catalase Activity

To measure the activity of the CAT enzyme in the serum of the studied animal, the Abei method was employed. In this method, a CAT substrate solution was prepared by mixing hydrogen peroxide (12 mM) and phosphate buffer (50 mM, pH 7.2). Subsequently, about 3 mL of CAT substrate solution and 50 μL of serum from the studied animal were mixed in a test tube, and the change in absorbance was evaluated spectrophotometrically at a wavelength of 240 nm every 30 seconds for 2 minutes against a blank (24).

### 3.9. Assessment of Superoxide Dismutase Activity

The Kono method was employed to measure the activity of the SOD enzyme in the serum of the studied rats. In this method, 2 mL of the SOD assay solution was mixed with 50 μL of hydroxylamine hydrochloride solution and 50 μL of the serum from the studied animal in a test tube. The rate of hydroxylamine autoxidation was then evaluated spectrophotometrically at 560 nm every 30 seconds for 2 minutes against a blank (25).

### 3.10. Nitric Oxide Level Assessment

To determine the level of nitric oxide (NO) in the serum of the studied rats, the Griess reagent and the colorimetric method were used. For this, equal volumes of rats’ serum samples (750 microliters) were mixed with the same volume of Griess reagent in a test tube. The resulting mixture was then incubated for 10 minutes at ambient temperature in the dark. The reaction mixture of nitrite with Griess reagent turns purple. The absorbance of the samples against the blank was then read at 540 nm (26).

### 3.11. Statistical Analysis

IBM SPSS Statistics software (version 21, USA) was employed for statistical analysis. One-way ANOVA and Tukey’s post-hoc tests were used to compare the biochemical values among the experimental groups. P-values less than 0.05 were considered statistically significant.

## 4. Results

Our study considered the treatment effects of the extract of black walnut in a methimazole-induced hypothyroidism model in rats. All rats survived until the end of the experiment.

### 4.1. Chemical Composition of the Walnut Oil

**Figure 1.**
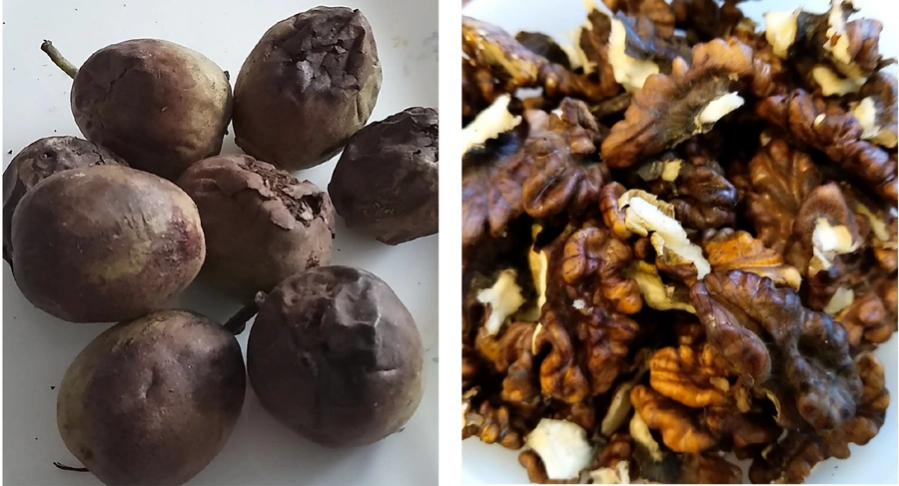
*Juglans nigra*, black walnut

**Figure 2.**
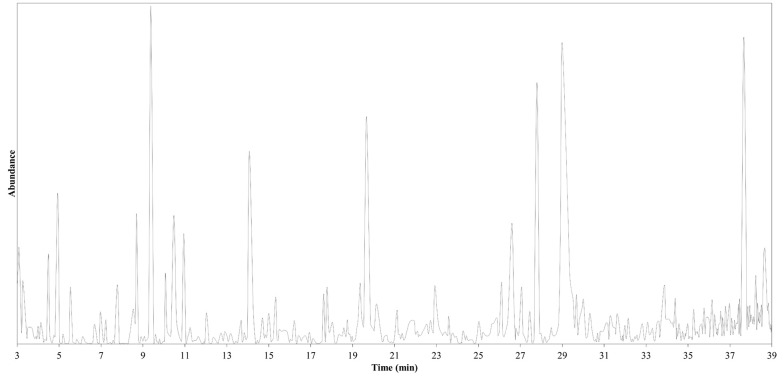
A gas chromatogram of the chemical elements in the hexane extract of walnut kernels; the X-axis represents compound abundance, and the Y-axis represents retention time.

To extract the oil from black walnut kernel (Figure 1), its powder was mixed well with EtOH at room temperature overnight. Then, the mixture was filtered off to obtain a clear extract. EtOH was removed slowly at 40°C using a rotary instrument under reduced pressure. The oily extract was then analyzed using a GC-Mass instrument. As shown in Figure 2, GC-MS analysis of an extract of black walnut kernels revealed a variety of phytochemical compounds.

**Figure 3.**
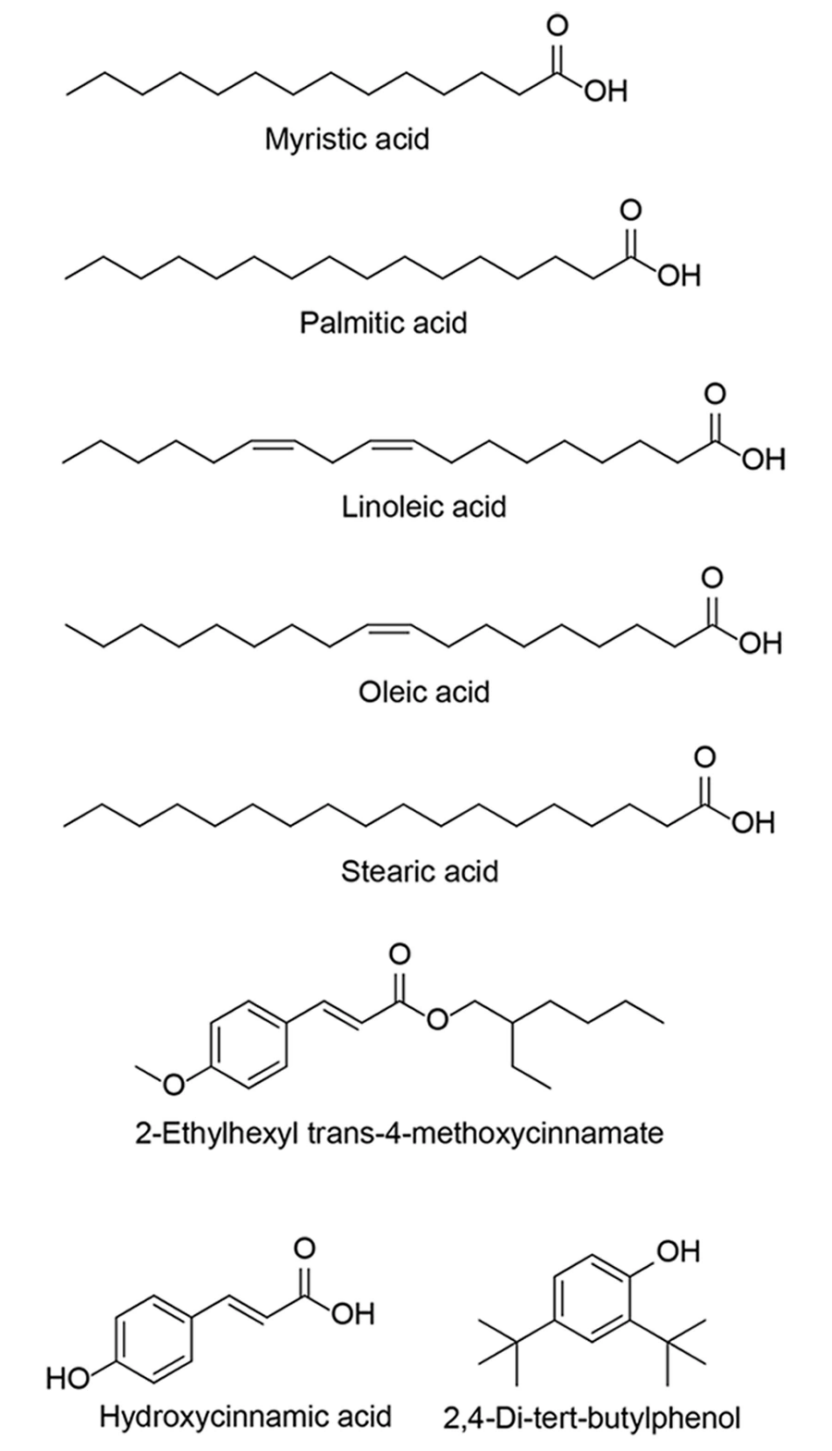
The structures of fatty acids and phenolic compounds found in *Juglans nigra* oil

Literature surveys reveal that linoleic acid (55 - 94%) and oleic acid (13 - 24%) are the main fatty acids in walnut oil (27). Considerable amounts of monounsaturated oleic acid and small amounts of saturated fatty acids are suitable for maintaining a healthy life, preventing cardiovascular diseases (28). Noticeably, polyunsaturated acids constitute the largest amount of walnut oil; however, palmitic acid was also found as the main saturated fatty acid in the range of 5 - 6% (29). GC-Mass analysis (Table 2, Figure 3) of the *J. nigra* oil exhibited the presence of linoleic acid in the highest percentage (21.2%), followed by oleic acid (17.9%), stearic acid (9.1%), palmitic acid (12.2%), and myristic acid (1.0%), respectively.

**Table 2.** Chemical Composition of Ethanolic Extract of Walnut Kernel

Chemical Categories (%)	Carbon Number	Content (%)	Reference Content (%)
**Fatty acids 63.4**			
Myristic acid	C14	1.0	0.02 - 1.9 (30, 31)
Palmitic acid or palmitate esters	C16	12.2	6.2 - 30.6 (27, 29, 30)
Linoleic acid	C18:2	21.2	57 - 64 (27, 30)
Oleic acid or ethyl oleate	C18:1	17.9	13 - 24 (27, 31)
Stearic acid or stearate esters	C18:0	9.1	2.7 - 31 (27, 30)
**Alkanes 17.8**			2 - 47 (32)
Hentriacontane	C31	0.4	
Octacosane	C28	0.4	
Heptacosane	C27	0.4	
Hexacosane	C26	0.8	
Pentacosane	C25	0.7	
Tetracosane	C24	0.4	
Tricosane	C23	0.7	
Docosane	C22	0.7	
Heneicosane	C21	0.7	
Eicosane	C20	0.6	
Nonadecane	C19	1.7	
Octadecane	C18	0.7	
Heptadecane	C17	1.2	
Hexadecane	C16	1.5	
Pentadecane	C15	2.7	
Tetradecane	C14	2.3	
Tridecane	C13	0.7	
Dodecane	C12	1.2	
**Cinnamic acid derivatives**			0.5 - 2.5
2-Ethylhexyl trans-4- methoxycinnamate	C18	0.5	
Hydrocinnamic acid	C9	4.3	
**Phenols 18.8**			2 - 68 (32, 33)
2,4-Di-tert-butylphenol	C14	8.3	
4-tert-Octylphenol	C14	2.4	

Various studies have revealed that walnut oil contains more than fifteen hydrocarbons, among which the main alkanes were C14 - C20. The species of walnut and the environment can affect the alkane composition (27). For instance, AbouRayya et al. discovered that nonadecane (30.07%) and octacosane (17.17%) constitute the main hydrocarbons in kernel oil of walnut trees growing in a private commercial region located in Egypt (30). Here, GC-Mass analysis shows the presence of various saturated hydrocarbons from dodecane (C12) to hentriacontane (C31), among which tetradecane, pentadecane, and nonadecane were predominant.

In 2022, Xu et al. found that phenolic ingredients of walnut can inhibit the growth of *Colletotrichum gloeosporioides*, showing anti-fungal activities (34). GC-Mass analysis could detect three kinds of phenolic compounds, including 2-ethylhexyl trans-4-methoxycinnamate, hydroxycinnamic acid, and 2,4-di-tert-butylphenol. Previously, 2,6-di-tert-butylphenol (42.9%) and 2,4-di-tert-butylphenol (1.0 - 68.0%) were extracted from Turkish walnut (32). In 2018, Persic et al. identified 16 phenolic compounds in the fresh kernel of walnut, among which hydroxycinnamic acid and quinic acid were the most abundant phenolic compounds (35). Hydroxycinnamic acid and its derivatives are bioactive natural compounds, exhibiting antioxidant, anticancer, antibacterial, and anti-inflammatory activities (36-38). It must be added that 2.3% of the walnut oil ingredients were not identifiable.

### 4.2. Phenolic and Flavonoid Content

Folin-Ciocalteu and aluminum chloride colorimetric procedures were utilized to measure the total phenolic levels and flavonoid contents of black walnut kernel oil. The results showed the oil contained 150 ± 8 µg of GAE per g of extract for phenolic compounds and 83.3 ± 5.5 µg of QUE per g of extract for flavonoid compounds, which are predictors of walnut kernel antioxidant capacity.

### 4.3. Thyroid Hormones

In our study on the effects of the ethanolic extract of Persian black walnut (Table 3), findings indicated that administration of this extract led to an increase in serum T4 hormone levels (6.12 ± 0.28, P > 0.05) compared to the methimazole-induced stress group (5.6 ± 0.35, P < 0.05) as well as the levothyroxine-treated stress group (5.6 ± 0.48, P < 0.05). This suggests that the ethanolic extract of black walnut was more effective than levothyroxine in improving thyroid function in methimazole-induced hypothyroidism. However, the differences were not statistically significant (P > 0.05).

**Table 3.** Biochemical Evaluation of Thyroid Hormone Profiles Following Black Walnut Extract Administration ^a, b^

Variables	Control	Control-Stress	Control-Walnut	Stress-Walnut	Stress-Levothyroxine
**T4 (nmol/L)**	6.06 ± 0.40	5.6 ± 0.35	5.68 ± 0.41	6.12 ± 0.28	5.6 ± 0.48
P-value	0.002	0.004	0.633	0.717	0.005
**T3 (nmol/L)**	0.74 ± 0.11	0.48 ± 0.08 ^c^	0.48 ± 0.10 ^c^	0.44 ± 0.15	0.52 ± 0.13
P-value	0.001	0.004	0.004	0.925	0.925
**TSH (ng/mL)**	1.85 ± 0.69	2.66 ± 0.30	1.43 ± 0.30	1.71 ± 0.39 $$	1.21 ± 0.47 $$$
P-value	0.000	0.034	0.632	0.011	0.000
**Anti-TPO (IU/mL)**	30.26 ± 3.97	32.88 ± 1.91	29.88 ± 5.26	31.14 ± 4.11	24.38 ± 6.36 $
P-value	0.029	0.405	1.000	0.733	0.014

Abbreviations: T4, thyroxine; T3, triiodothyronine; TSH, thyroid-stimulating hormone; anti-TPO, thyroid peroxidase antibody.

^a^ Values are expressed as mean ± SD.

^b^ If the difference between the means is not statistically significant (P > 0.05), it is meaningless and no symbol is used.

^c^ This sign indicates a comparison with the control group. The difference between the means of two treatments is significant at P < 0.05.

Regarding the effect of the extract on serum T3 hormone levels, our results showed that the methimazole-treated stress group exhibited a significant decrease in T3 levels compared to the control group (0.48 ± 0.08, P < 0.05). Furthermore, in the methimazole-stressed group that received the black walnut extract, the extract did not significantly restore T3 levels (0.44 ± 0.15, P > 0.05) and was less effective than levothyroxine in enhancing T3 levels compared to both the stress and control groups. Given that T3 is primarily produced in the liver through the peripheral conversion of T4, it is likely that rats exposed to methimazole experienced some degree of hepatic impairment or toxicity, either from the drug itself or the extract. This may have hindered the conversion of T4 to T3, leading to unchanged or suboptimal T3 levels despite extract administration. Therefore, it can be inferred that the lack of improvement in T3 levels is likely due to impaired liver function in this process. Furthermore, the TSH and anti-TPO levels were increased in the methimazole-treated stress group in comparison with the control group (P > 0.05).

### 4.4. Assessment of Oxidant Status

Despite the presumed antioxidant effect, oxidative stress markers (Table 4) such as NO and MDA showed an increase. This can be explained by the fact that in our comparisons, the stressed group was compared to the control group, while the extract-treated stressed groups were compared to the methimazole-treated stress group. Additionally, the extract-treated stress group was also compared to the levothyroxine-treated stress group. The MDA and NO concentrations were measured to determine the amount of reactive oxygen species (ROS). Consequently, using treatment with black walnut kernel extract in hypothyroidism rats, the NO and MDA levels were increased in the methimazole-treated stress group in comparison with the control group (P > 0.05). The concentrations of NO and MDA in rats from the methimazole-stress group treated with black walnut extract were reduced compared to the methimazole-stress group (P > 0.05). Similarly, the concentration of NO in the methimazole-stress group treated with levothyroxine was also reduced compared to the methimazole-stress group (P > 0.05). Furthermore, the concentration of MDA in the methimazole-stress group treated with levothyroxine showed a significant reduction compared to the methimazole-stress group (P < 0.001).

**Table 4.** Oxidative Stress Markers Include Malondialdehyde and Nitric Oxide ^a, b^

Variables	Control	Control-Stress	Control-Walnut	Stress-Walnut	Stress-Levothyroxine
**MDA (µmol/L)**	2.93 ± 0.14	3.39 ± 0.35	2.46 ± 0.33	2.97 ± 0.31	2.46 ± 0.10 $$$
P-value	0.000	0.180	0.112	0.519	0.000
**NO (µmol/L)**	26.18 ± 3.32	30.46 ± 1.77	26.53 ± 1.15	25.25 ± 4.08	24.18 ± 3.3
P-value	0.014	0.132	0.997	0.028	0.012

Abbreviation: MDA, malondialdehyde.

^a^ Values are expressed as mean ± SD.

^b^ If the difference between the means is not statistically significant (P > 0.05), it is meaningless and no symbol is used.

Despite the anticipated antioxidant properties of the tested extract (next section), the results demonstrated an unexpected increase in the levels of oxidative stress biomarkers, namely MDA and NO. Several possible explanations may account for this observation, involving the effect of time on the antioxidant activities of the extract, dose-dependent effects, or assay limitations. Further studies using varying doses and time points are necessary to fully understand the redox-modulating properties of the extract.

### 4.5. Assessment of Antioxidant Status

The CAT and SOD (Table 5) were measured to determine the body’s antioxidant capacity. Consequently, using treatment with black walnut kernel extract in hypothyroidism rats, the SOD level was decreased in the methimazole-treated stress group in comparison with the control group (P > 0.05), while the CAT level was significantly decreased in the methimazole-treated stress group in comparison with the control group (P < 0.05).

**Table 5.** Antioxidant Factors Including Catalase and Superoxide Dismutase ^a^

Variables	Control	Control-Stress	Control-Walnut	Stress-Walnut	Stress-Levothyroxine
**SOD (U/mL)**	0.388 ± 0.008	0.37 ± 0.015	0.4 ± 0.01	0.374 ± 0.018	0.392 ± 0.019
P-value	0.407	0.673	0.948	0.999	0.453
**CAT (U/mg)**	108.77 ± 5.15	91.51 ± 8.39 ^b^	103.4 ± 6.38	93.19 ± 10.17	96.73 ± 8.10
P-value	0.014	0.040	1.000	0.987	0.487

Abbreviations: SOD, superoxide dismutase; CAT, catalase.

^a^ Values are expressed as mean ± SD.

^b^ A comparison with the control group. The difference between the means of two treatments is significant at P > 0.05..

The concentrations of SOD and CAT in rats from the methimazole-stress group treated with black walnut extract increased compared to the methimazole-stress group (P > 0.05). Similarly, the concentrations of SOD and CAT in the methimazole-stress group treated with levothyroxine also increased compared to the methimazole-stress group (P > 0.05).

### 4.6. Histopathological Analysis

**Figure 4.**
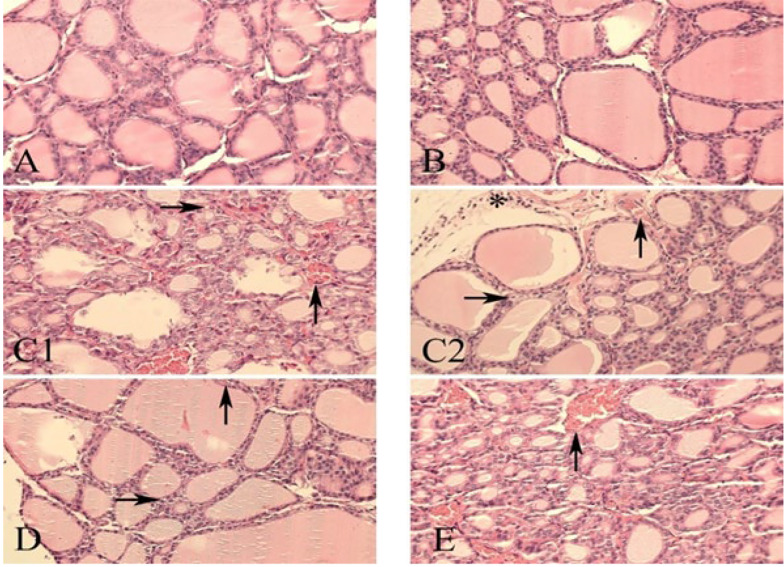
Thyroid tissue: A, control group showing normal thyroid tissue architecture; B, black walnut extract group showing normal histological appearance; C, methimazole group exhibiting vascular congestion (↑), vacuolar degeneration (→), and inflammatory cell infiltration (*); D, methimazole + black walnut extract group showing vascular congestion (↑) and vacuolar degeneration (→), with no inflammatory infiltration; E, methimazole + levothyroxine group showing vascular congestion (↑), with no other pathological findings (abbreviation: H&E, hematoxylin and eosin staining; magnification 40x).

In the assessment of thyroid tissue, qualitative evaluation of pathological lesions was performed. The absence of lesions was recorded as negative. A scoring system was used: 1+ indicated mild lesions, 2+ moderate lesions, and 3+ (which was not observed in this study) indicated severe lesions (Table 6). Three main pathological changes were evaluated: Congestion, vacuolar degeneration, and infiltration of inflammatory cells (Figure 4). In the control group (Figure 4A), no pathological lesions were observed; there was no congestion, vacuolar degeneration, or inflammatory cell infiltration. Similarly, in the group treated with black walnut extract alone, no lesions were detected.

**Table 6.** Thyroid Tissue

Variables	Inflammatory Cell Infiltration	Vacuolar Degeneration	Congestion
**Control**	-	-	-
**Walnut extract**	-	-	-
**Methimazole**	+	+	++
**Methimazole + Walnut extract**	-	+	+
**Methimazole + Levothyroxine**	-	-	++

In the methimazole-treated group, moderate congestion was observed, along with mild vacuolar degeneration and mild infiltration of inflammatory cells. This suggests a dose-response effect of methimazole. In the group receiving both methimazole and black walnut extract, mild congestion and mild vacuolar degeneration were observed, but no infiltration of inflammatory cells was detected. These findings suggest that black walnut extract may exert anti-inflammatory effects by reducing inflammatory cell infiltration. Moreover, it also reduced congestion, although it did not show any significant effect on vacuolar degeneration. In the group treated with both methimazole and levothyroxine, no change in congestion was observed compared to the methimazole-only group (i.e., moderate congestion), while vacuolar degeneration and inflammatory cell infiltration were not observed. In the histological images, upward arrows indicate vascular congestion, rightward arrows indicate vacuolar degeneration, and asterisks represent inflammatory cells.

## 5. Discussion

Hypothyroidism is one of the most important endocrine gland diseases, leading to the dysfunction of the thyroid hormones. We evaluated the effect of an oily extract of black walnut (*J. nigra*) kernels on methimazole-induced hypothyroidism in adult Wistar rats.

In the study by Sharafati Chaleshtori et al., walnut kernel extract exhibited antimicrobial effects against three bacteria, making it a potential food preservative. The total phenol content of this extract was 365 ± 14.17 mg/g gallic acid equivalent, and its total flavonoid content was 285 ± 12.25 mg/g (39). According to our findings, the phenolic content of black walnut kernel was 150 ± 8 µg gallic acid per gram of extract, while its total flavonoid content was 83.3 ± 5.5 µg quercetin per gram of extract.

In the study by Mirazi et al., it was found that the ethanolic extract of sage (*Salvia*) could influence thyroid function by increasing thyroid hormone levels in hypothyroid rats. The ethanolic extract of certain plants, such as sage, stimulates the thyroid due to its anti-peroxidase and antioxidant properties, leading to elevated concentrations of T3 and T4 (40). Based on our results, the ethanolic extract of black walnut did not cause a significant difference in blood T4 levels in the studied rats (P > 0.05). However, regarding T3, black walnut extract led to a decrease in its concentration compared to the control group (P < 0.05).

On the other hand, Zabihi et al. found that the ethanolic extract of *Teucrium polium* increased serum thyroid hormone levels and reduced TSH levels (41). Our study revealed that the extract of black walnut increased TSH levels in rats exposed to methimazole-induced stress compared to the control group (P > 0.05), which contrasts with their findings.

**Figure 5.**
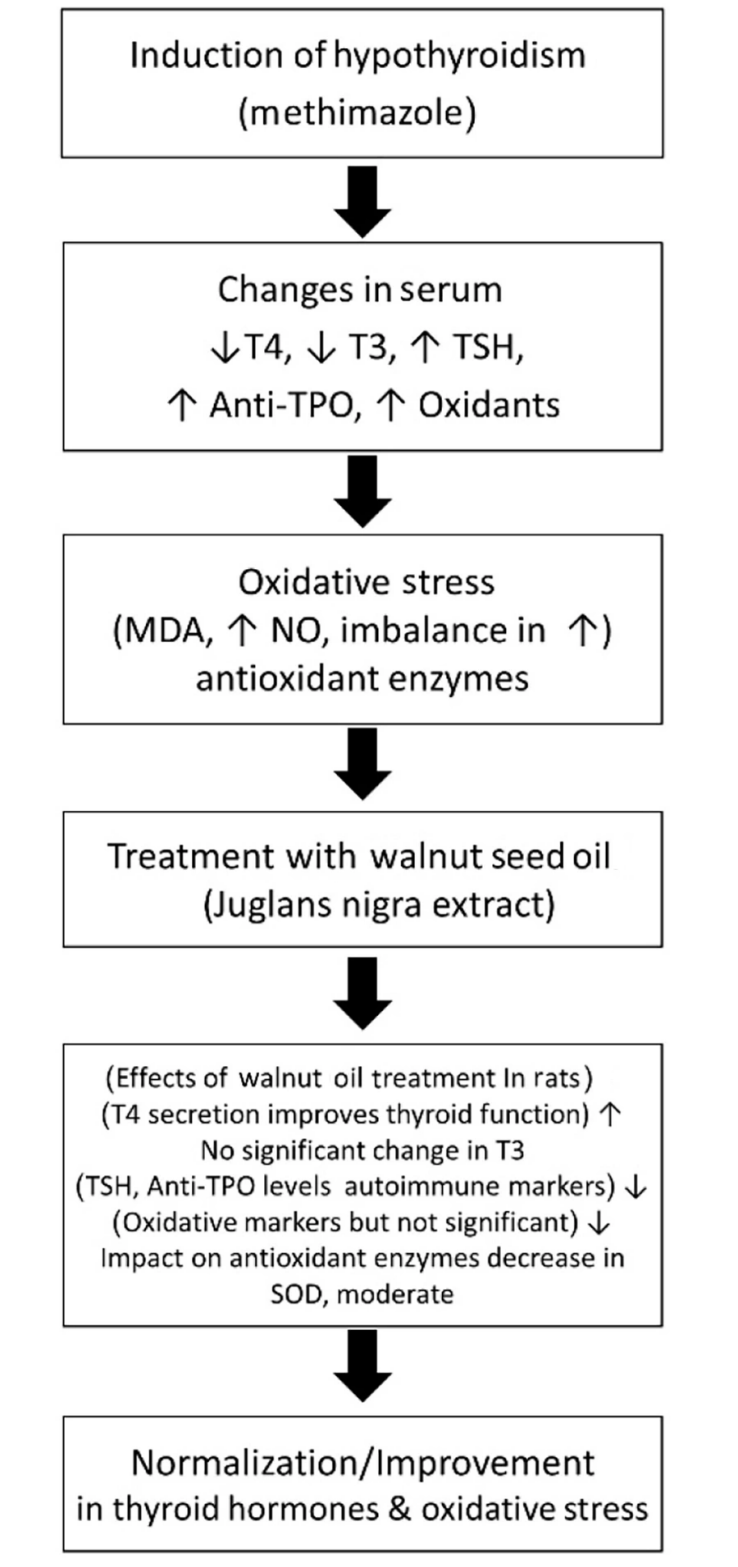
Experimental design

Overall, TPO antibodies play a crucial role in tissue damage associated with hypothyroidism caused by Hashimoto’s thyroiditis and atrophic thyroiditis. The cytotoxic effects of these antibodies on thyroid cells are attributed to their ability to fix complement, which accelerates thyroid dysfunction (42). Bhakat et al. found that vitamin D deficiency is a risk factor for Hashimoto’s thyroiditis. Their study demonstrated that vitamin D supplementation reduces thyroid autoimmunity, indicating a positive effect on both thyroid function and autoimmunity (43). In our research (Figure 5), the extract of black walnut had no significant effect on anti-TPO levels in rats subjected to methimazole-induced stress compared to the control group (P > 0.05).

Under normal conditions, a certain amount of free radicals is generated as a result of cellular activity and metabolism. These radicals are neutralized by antioxidants such as SOD and CAT in the body, maintaining a balance between oxidative stress and antioxidant defense. Any factor that disrupts this balance — either by increasing free radical production or reducing antioxidants — ultimately leads to oxidative stress, which in turn can contribute to pathological changes in the body (44). Scientific literature indicates that thyroid hormones play a vital role in numerous physiological processes, including oxidative metabolism regulation and mitochondrial respiration enhancement, which results in increased ROS production. Previous studies have reported that both hyperthyroidism and hypothyroidism are associated with oxidative stress and cellular damage (45, 46). In the study by Okays et al., it was observed that SOD and CAT levels were significantly reduced in the hypothyroid group compared to the control group. Hypothyroidism alters the oxidant-antioxidant balance in serum and tissues (47). Based on our findings, the extract of black walnut had no significant effect on SOD levels (P > 0.05), but it significantly reduced CAT levels in methimazole-stressed rats compared to the control group (P < 0.05).

Baskol et al. reported that increased ROS levels in hypothyroidism may create a pro-oxidative environment, leading to elevated MDA and NO levels. Consequently, lipid peroxidation may contribute to the pathogenesis of atherosclerosis in hypothyroidism (8). In our study, the extract of black walnut increased the levels of oxidative markers MDA and NO in methimazole-stressed rats compared to the control group (P > 0.05).

On the other hand, thyroid hormones have been shown in previous studies to increase NO production via the phosphatidylinositol 3-kinase/protein kinase B (PI3K/Akt) signal transduction pathway (48). Because NO is an oxidized nitrogen species, it has been used as an oxidative stress marker in several diseases. Numerous studies with contradictory findings have investigated the relationship between NO and thyroid disorders. For example, Mehrazin et al. discovered that hypothyroidism reduced the formation of NO metabolites in serum samples and the aortic wall of rats with propylthiouracil-induced fetal hypothyroidism (49). Likewise, Kumar et al. found that NO concentrations were significantly lower in hypothyroid subjects (50). Nevertheless, Verma et al. discovered that hypothyroid patients have considerably higher NO concentrations compared to healthy subjects (51).

Despite the well-documented antioxidant properties of walnut extract, mainly attributed to its phenolic compounds and unsaturated fatty acids, our findings revealed a paradoxical increase in oxidative stress markers, particularly MDA and NO, in the methimazole-stressed rats treated with walnut extract.

This observation contradicts the hypothesis of antioxidant protection and raises several mechanistic questions. One potential explanation is that the dose of walnut extract used (1000 mg/kg) may have been insufficient or inappropriate under the oxidative conditions induced by methimazole. Alternatively, the extract’s high polyunsaturated fatty acid content (especially linoleic and oleic acids) may have increased lipid peroxidation substrates, resulting in elevated MDA formation despite the presence of antioxidant phenolics.

Regarding NO, although NO can act as a signaling molecule with vasodilatory and cytoprotective roles, its overproduction, especially in the absence of adequate scavenging mechanisms, can lead to nitrosative stress. The observed increase in NO levels in this study may reflect an imbalance in NO synthase activity or an inflammatory response secondary to methimazole-induced tissue injury.

Furthermore, it is possible that the bioavailability and metabolic fate of walnut extract components were compromised in hypothyroid rats. Hypothyroidism is associated with reduced hepatic clearance, altered enzyme activity, and mitochondrial dysfunction, all of which could affect both oxidative metabolism and the pharmacodynamics of plant-derived compounds.

Despite the well-established antioxidant properties of black walnut, our findings showed a paradoxical increase in oxidative stress markers MDA and NO in methimazole-treated rats. This outcome may be attributed to several possible factors. First, the extract dosage may not have been sufficient to counteract methimazole-induced oxidative stress, or conversely, might have introduced additional oxidative substrates due to its high polyunsaturated fatty acid content. Second, the duration of treatment (6 weeks) may not have allowed for full antioxidant responses to manifest. Third, the antioxidant activity of plant-based extracts is known to be context-dependent, varying with the metabolic and inflammatory status of the host. Additionally, assay limitations and lack of tissue-level measurements (e.g., lipid peroxidation in thyroid or liver tissues) may have affected the interpretation of oxidative stress outcomes. These limitations should be addressed in future studies using a broader range of doses, time points, and molecular oxidative biomarkers to better characterize the redox effects of black walnut extract under hypothyroid conditions.

### 5.1. Conclusions

While the ethanolic extract of black walnut showed a trend toward increased T4 levels, the change was not statistically significant, and T3 levels were significantly decreased. Therefore, no definitive improvement in thyroid function was observed. Future studies are required to further clarify these effects. Our study has some limitations. First, liver enzymes were not examined in the current study. Also, we did not assess non-enzymatic antioxidants such as retinol (vitamin A) and alpha-tocopherol. Moreover, the presence of trace elements such as iodine, which is important for thyroid hormone synthesis, was not investigated in our research. Therefore, these limitations need to be included in future studies. In addition, we proposed that future research investigate the effects of walnut leaf extract on hypothyroidism. It is also recommended to consider the effect of walnut kernel extract on hyperthyroidism. In summary, these findings highlight the complexity of plant-based antioxidant therapy in endocrine disorders and emphasize the need for comprehensive mechanistic studies that include tissue-level oxidative damage assays, time-course analyses, and metabolic profiling of extract constituents.

## Data Availability

The datasets used and/or analyzed during the current study are available from the corresponding author upon reasonable request.

## References

[1] Hegedus L, Bianco AC, Jonklaas J, Pearce SH, Weetman AP, Perros P (2022). Primary hypothyroidism and quality of life.. Nat Rev Endocrinol..

[2] Jansen HI, Boelen A, Heijboer AC, Bruinstroop E, Fliers E (2023). Hypothyroidism: The difficulty in attributing symptoms to their underlying cause.. Front Endocrinol (Lausanne)..

[3] Sorensen JR, Winther KH, Bonnema SJ, Godballe C, Hegedus L (2016). Respiratory Manifestations of Hypothyroidism: A Systematic Review.. Thyroid..

[4] Hulbert AJ (2000). Thyroid hormones and their effects: a new perspective.. Biol Rev Camb Philos Soc..

[5] Sinha RA, Yen PM (2024). Metabolic Messengers: Thyroid Hormones.. Nat Metab..

[6] Roy G, Mugesh G (2005). Anti-thyroid drugs and thyroid hormone synthesis: effect of methimazole derivatives on peroxidase-catalyzed reactions.. J Am Chem Soc..

[7] Halliwell B (2024). Understanding mechanisms of antioxidant action in health and disease.. Nat Rev Mol Cell Biol..

[8] Baskol G, Atmaca H, Tanriverdi F, Baskol M, Kocer D, Bayram F (2007). Oxidative stress and enzymatic antioxidant status in patients with hypothyroidism before and after treatment.. Exp Clin Endocrinol Diabetes..

[9] Chakrabarti SK, Ghosh S, Banerjee S, Mukherjee S, Chowdhury S (2016). Oxidative stress in hypothyroid patients and the role of antioxidant supplementation.. Indian J Endocrinol Metab..

[10] Pisoschi AM, Pop A (2015). The role of antioxidants in the chemistry of oxidative stress: A review.. Eur J Med Chem..

[11] Floyd RA (1999). Antioxidants, oxidative stress, and degenerative neurological disorders.. Proc Soc Exp Biol Med..

[12] Quratul A, Shadab M, Siddiqui MB, Ansari MA, Shoaib S, Islam N (2024). Medicinal and Nutritional Importance of Juglans regia Linn. on Human Health.. Medicinal Plants and their Bioactive Compounds in Human Health: Volume 1..

[13] Chudhary Z, Khera RA, Hanif MA, Ayub MA, Hamrouni L, Hanif MA, Nawaz H, Mumtaz K, Byrne HJ (2020). Walnut.. Medicinal Plants of South Asia..

[14] Adelakun SA, Ukwenya VO, Ogunlade BS, Aniah JA, Ibiayo AG (2018). Nitrite-induced testicular toxicity in rats: therapeutic potential of walnut oil.. JBRA Assisted Reproduction..

[15] Câmara CRS, Schlegel V (2016). A Review on the Potential Human Health Benefits of the Black Walnut: A Comparison with the English Walnuts and Other Tree Nuts.. Int J Food Properties..

[16] Vu DC, Nguyen TH, Ho TL (2020). An overview of phytochemicals and potential health-promoting properties of black walnut.. RSC Advances..

[17] Ho KV, Lei Z, Sumner LW, Coggeshall MV, Hsieh HY, Stewart GC (2018). Identifying Antibacterial Compounds in Black Walnuts (Juglans nigra) Using a Metabolomics Approach.. Metabolites..

[18] Amarowicz R, Dykes GA, Pegg RB (2008). Antibacterial activity of tannin constituents from Phaseolus vulgaris, Fagoypyrum esculentum, Corylus avellana and Juglans nigra.. Fitoterapia..

[19] Ainsworth EA, Gillespie KM (2007). Estimation of total phenolic content and other oxidation substrates in plant tissues using Folin-Ciocalteu reagent.. Nat Protoc..

[20] Uribe E, Pasten A, Lemus-Mondaca R, Vega-Gálvez A, Quispe-Fuentes I, Ortiz J (2015). Comparison of Chemical Composition, Bioactive Compounds and Antioxidant Activity of Three Olive-Waste Cakes.. J Food Biochem..

[21] Honari N, Pouraboli I (2019). [The Effect of Hydroalcoholic Extract of Thymus caramanicus on Serum Testosterone and Testis Antioxidant Enzymes Levels in Streptozotocin Induced Diabetic Rats: An Experimental Study].. J Rafsanjan Univ Med Sci..

[22] Agharanya JC (1990). Clinical usefulness of ELISA technique in the assessment of thyroid function.. West Afr J Med..

[23] Atmakusuma TD, Nasution IR, Sutandyo N (2021). Oxidative Stress (Malondialdehyde) in Adults Beta-Thalassemia Major and Intermedia: Comparison Between Before and After Blood Transfusion and Its Correlation with Iron Overload.. Int J Gen Med..

[24] Aebi H, Bergmeyer HU (1974). Catalase.. Methods of Enzymatic Analysis..

[25] Kono Y (1978). Generation of superoxide radical during autoxidation of hydroxylamine and an assay for superoxide dismutase.. Arch Biochem Biophys..

[26] Kharazmi K, Heydari A, Ardjmand A (2019). [The effect of curcumin pre-treatment on morphine-induced inhibitory memory impairment and nitric oxide level in rat].. Feyz Med Sci J..

[27] Martínez ML, Mattea MA, Maestri DM (2006). Varietal and crop year effects on lipid composition of walnut (Juglans regia) genotypes.. J Am Oil Chem' Soc..

[28] Lu Y, Zhao J, Xin Q, Yuan R, Miao Y, Yang M (2024). Protective effects of oleic acid and polyphenols in extra virgin olive oil on cardiovascular diseases.. Food Sci Hum Wellness..

[29] Li L, Tsao R, Yang R, Kramer JK, Hernandez M (2007). Fatty acid profiles, tocopherol contents, and antioxidant activities of heartnut (Juglans ailanthifolia Var. cordiformis) and Persian walnut (Juglans regia L.).. J Agric Food Chem..

[30] AbouRayya S, Kasem N, Ibrahim E, Mahmoud T, Salim R (2021). Fruit physical, chemical and molecular identification of three walnuts (Juglans regia L.) genotypes grown in Egypt.. Egypt J Chem..

[31] Kesen S, Amanpour A, Selli S (2018). Comparative evaluation of the fatty acids and aroma compounds in selected Iranian nut oils.. Europ J Lipid Sci Technol..

[32] Şirin N, Coşkun NC, Adem Ş (2024). Effects of Walnut Septum on The Enzyme Pathways Associated with Plasma Cholesterol Level.. Konuralp Tıp Dergisi..

[33] Bakkalbaşı E (2018). Oxidative stability of enriched walnut oil with phenolic extracts from walnut press-cake under accelerated oxidation conditions and the effect of ultrasound treatment.. J Food Measur Characterization..

[34] Xu H, Wang G, Zhang J, Zhang M, Fu M, Xiang K (2022). Identification of phenolic compounds and active antifungal ingredients of walnut in response to anthracnose (Colletotrichum gloeosporioides).. Postharvest Biol Technol..

[35] Persic M, Mikulic-Petkovsek M, Slatnar A, Solar A, Veberic R (2018). Changes in phenolic profiles of red-colored pellicle walnut and hazelnut kernel during ripening.. Food Chem..

[36] Coman V, Vodnar DC (2020). Hydroxycinnamic acids and human health: recent advances.. J Sci Food Agric..

[37] Teixeira J, Gaspar A, Garrido EM, Garrido J, Borges F (2013). Hydroxycinnamic acid antioxidants: an electrochemical overview.. Biomed Res Int..

[38] Rocha LD, Monteiro MC, Teodoro AJ (2012). Anticancer properties of hydroxycinnamic acids-a review.. Cancer Clin Oncol..

[39] Sharafati Chaleshtori R, Chaleshtori FS, Rafieian M (2011). Biological characterization of Iranian walnut (Juglans regia) leaves.. Turkish J Biol..

[40] Mirazi N, Abdolmaleki N, Mahmoodi M (2013). [Study of Salvia Officinalis Hydroethanolic Extract on Serum Thyroid Hormone Levels in Hypothyroid Male Rat].. Avicenna J Clin Med..

[41] Zabihi E, Motavallibashi SE, Panahpour H, Sheikhkanloui Milan H (2019). Effect of Hydroalcoholic Extract of Truffle (Terfezia Boudieri) on Serum Level of Thyroid Hormones in Male Rats.. J Arak Univ Med Sci..

[42] Mariotti S, Caturegli P, Piccolo P, Barbesino G, Pinchera A (1990). Antithyroid peroxidase autoantibodies in thyroid diseases.. J Clin Endocrinol Metab..

[43] Bhakat B, Pal J, Das S, Charaborty SK, SircarMedical NR, Kolkata (2023). A Prospective Study to Evaluate the Possible Role of Cholecalciferol Supplementation on Autoimmunity in Hashimoto's Thyroiditis.. J Assoc Physicians India..

[44] Ighodaro OM, Akinloye OA (2019). First line defence antioxidants-superoxide dismutase (SOD), catalase (CAT) and glutathione peroxidase (GPX): Their fundamental role in the entire antioxidant defence grid.. Alexandria J Med..

[45] Weitzel JM, Iwen KA, Seitz HJ (2003). Regulation of mitochondrial biogenesis by thyroid hormone.. Exp Physiol..

[46] Goglia F, Silvestri E, Lanni A (2002). Thyroid hormones and mitochondria.. Biosci Rep..

[47] Oktay S, Uslu L, Emekli N (2017). Effects of altered thyroid states on oxidative stress parameters in rats.. J Basic Clin Physiol Pharmacol..

[48] Carrillo-Sepulveda MA, Ceravolo GS, Fortes ZB, Carvalho MH, Tostes RC, Laurindo FR (2010). Thyroid hormone stimulates NO production via activation of the PI3K/Akt pathway in vascular myocytes.. Cardiovasc Res..

[49] Mehrazin F, Ghasemi A, Shiravi A, Zahediasl S (2013). [Effect of L-arginine on serum and tissue levels of nitric oxide metabolites in offsprings of rats with gestational hypothyroidism].. Pejouhesh dar Pezeshki (Res Med)..

[50] Kumar A, Suresh DR, Annam V, Srikrishna R (2012). Significance of early biochemical markers of atherosclerosis in subclinical hypothyroidism patients with normal lipid profile.. Int J Biol Med Res..

[51] Verma M, Dahiya K, Ghalaut V, Seth S, Roy P, Basu A (2015). Thyroid disorders and nitric oxide levels.. J Sci Med..

